# Behavioral relevance of species-specific vasotocin anatomy in gregarious finches

**DOI:** 10.3389/fnins.2013.00242

**Published:** 2013-12-17

**Authors:** Aubrey M. Kelly, James L. Goodson

**Affiliations:** Department of Biology, Indiana UniversityBloomington, IN, USA

**Keywords:** affiliation, sociality, songbird, anxiety, medial bed nucleus of the stria terminalis, vasopressin, *Uraeginthus angolensis*, sex differences

## Abstract

Despite substantial species differences in the vasotocin/vasopressin (VT/VP) circuitry of the medial bed nucleus of the stria terminalis (BSTm) and lateral septum (LS; a primary projection target of BSTm VT/VP cells), functional consequences of this variation are poorly known. Previous experiments in the highly gregarious zebra finch (Estrildidae: *Taeniopygia guttata*) demonstrate that BSTm VT neurons promote gregariousness in a male-specific manner and reduce anxiety in both sexes. However, in contrast to the zebra finch, the less gregarious Angolan blue waxbill (Estrildidae: *Uraeginthus angolensis*) exhibits fewer VT-immunoreactive cells in the BSTm as well as differences in receptor distribution across the LS subnuclei, suggesting that knockdown of VT production in the BSTm would produce behavioral effects in Angolan blue waxbills that are distinct from zebra finches. Thus, we here quantified social contact, gregariousness (i.e., preference for the larger of two groups), and anxiety-like behavior following bilateral antisense knockdown of VT production in the BSTm of male and female Angolan blue waxbills. We find that BSTm VT neurons promote social contact, but not gregariousness (as in male zebra finches), and that antisense effects on social contact are significantly stronger in male waxbills than in females. Knockdown of BSTm VT production has no effect on anxiety-like behavior. These data provide novel evidence that species differences in the VT/VP circuitry arising in the BSTm are accompanied by species-specific effects on affiliation behaviors.

## Introduction

In most tetrapod species, the vasotocin/vasopressin (VT/VP) circuitry of the medial bed nucleus of the stria terminalis (BSTm) and lateral septum (LS) is seasonally variable, regulated by steroid hormones, and sexually differentiated (males > females)—often to an extreme degree in each of these respects (Goodson and Bass, 2001; De Vries and Panzica, 2006; Goodson and Thompson, 2010). This circuitry is absent in fishes (Goodson and Bass, 2000; Goodson et al., 2003; Greenwood et al., 2008), but is present in virtually all tetrapods, suggesting the presence of conserved behavioral functions. As reviewed below, these conserved functions appear to include a variety of affiliation functions. However, substantial species differences are observed in the numbers of VT/VP cells in the BSTm, and in the densities and topographical distributions of receptors across LS subnuclei, suggesting the presence of functional differences as well (e.g., see Insel et al., 1991; Wang, 1995; Bester-Meredith et al., 1999; Goodson et al., 2009b; Kabelik et al., 2010).

Importantly, although VT/VP cells of the BSTm exhibit direct projections to the ventral hippocampus, medial preoptic nucleus, periaqueductal gray, lateral habenula, and LS (De Vries and Al-Shamma, 1990; Absil et al., 2002; De Vries and Panzica, 2006; Goodson and Thompson, 2010), each of these sites likely also receives peptide in a paracrine manner from other VT/VP cell groups. For instance, magnocellular neurons of the hypothalamus release peptide volumetrically from soma and dendrites (Landgraf and Neumann, 2004; Ludwig and Leng, 2006), and PVN VP neurons modulate LS physiology in the absence of direct innervation (Disturnal et al., 1986). Indeed, binding of endogenous VP in the LS modulates behavior in Syrian hamsters (*Mesocricetus auratus*) (e.g., Irvin et al., 1990; Albers and Cooper, 1995) despite the fact that this species exhibits a complete lack of VP cells in the BSTm (Bolborea et al., 2010). Given these observations, it is difficult to link peptide effects in any given target zone specifically to one VT/VP cell group or another, and thus direct manipulations of the cells are required to establish function.

To date, the majority of evidence regarding behavioral functions of BSTm VT/VP neurons has come from immediate early gene studies, which show that these neurons respond primarily to affiliation-related stimuli, particularly in males and in the context of reproduction. For example, BSTm VT/VP cells increase their transcriptional activity (as measured by Fos induction, a proxy marker of neural activity) selectively in response to (1) positive but not negative social stimuli in a variety of estrildid finch species (Goodson and Wang, 2006); (2) copulation but not aggressive interactions in mice (Ho et al., 2010); and (3) appetitive sexual behavior but not agonistic behavior in chickens (Xie et al., 2011). The percent of BSTm VT cells expressing Fos also correlates with the intensity of male sexual behavior in brown anoles (*Anolis sagrei*), but not with the intensity of male-male aggression (Kabelik et al., 2013). Thus, VT/VP neurons of the BSTm appear to be sensitive to social valence, and in fact, these neurons are differentially responsive to same-sex stimuli in flocking and territorial finches (Goodson and Wang, 2006). Additionally, male zebra finches (*Taeniopygia guttata*) that reliably court females have significantly more VT-immunoreactive (-ir) neurons in the BSTm than males that fail to court females (Goodson et al., 2009a), and overnight cohabitation with a female increases VP mRNA in the BSTm of male prairie voles (*Microtus ochrogaster*) (Wang et al., 1994).

Consistent with the affiliation-related responses of the BSTm VT/VP neurons, we recently showed that antisense knockdown of VT production in these cells increases same-sex aggression in male zebra finches, but not females, and reduces courtship singing in males (Kelly and Goodson, 2013). These experiments suggest the hypothesis that the phylogenetically widespread sex difference in BSTm VT/VP production serves to promote male affiliative behavior in a context that is tied to reproduction, while concomitantly offsetting the tendency for males to be more aggressive than females (Kelly and Goodson, 2013). This hypothesis is strongly supported by a variety of other data. For instance, less aggressive (long attack latency) male mice show a denser VP-ir innervation of the LS and more VP-ir neurons in the BSTm than do more aggressive males that exhibit short attack latencies (Compaan et al., 1993). Similarly, in sparrows, the density of VT immunolabeling in the BSTm correlates negatively with both individual and species differences in aggression (Goodson et al., 2012c), and infusions of VT into the LS decrease resident-intruder aggression in both sparrows and finches (Goodson, 1998a,b). Because VT/VP production in the BSTm is typically observed only during the breeding season, these findings suggest that VT/VP circuitry of the BSTm-LS modulates male behavior primarily in the context of reproduction.

In addition to reproductive functions, BSTm VT neurons also promote gregariousness in male zebra finches, and modulate anxiety in both males and females (Kelly et al., 2011). However, unlike seasonally breeding bird species, opportunistically breeding finch species do not exhibit seasonal fluctuation in VT-ir cell numbers in the BSTm, and VT-ir cell numbers in adult zebra finches are not regulated by steroid hormones (Kabelik et al., 2010). Thus, the involvement of BSTm VT neurons in non-reproductive behaviors such as grouping likely evolved after the evolutionary loss of seasonal reproduction and seasonal VT expression, effectively allowing VT circuitry that modulates reproductive affiliation to be co-opted for non-reproductive aspects of affiliation, as well (Goodson, 2013). Notably, available evidence suggests that VP production in the human BSTm may likewise be uncoupled from reproductive state, given that VP neurons have even been detected in a post-menopausal woman receiving anti-estrogen treatment (Fliers et al., 1986), suggesting the possibility of functional parallels between humans and opportunistic finches (see Rilling et al., 2012; Goodson, 2013).

Although the findings reviewed above suggest that the anatomy and functions of the BSTm-LS circuitry are highly conserved and are strongly tied to reproduction, there is nonetheless variation among species in VT/VP cell numbers in the BSTm and in the densities of V1a and OT receptors (V1aRs and OTRs; which tend to bind promiscuously) in the LS. For example, the monogamous mouse, *Peromyscus californicus*, exhibits more VP-ir labeling in the BSTm and greater VP receptor densities in the LS than the polygamous mouse, *Peromyscus leucopus* (Bester-Meredith et al., 1999), and conversely, the monogamous prairie vole exhibits fewer VP-ir cells in the BSTm but a higher density of VP-ir fibers in the LS than the polygamous meadow vole (*Microtus pennsylvanicus*) (Wang, 1995).

BSTm cell numbers and peptide binding sites also differ across finch species, and importantly, this variation mirrors convergent and divergent patterns of social evolution (Goodson et al., 2012a). Relevant studies have used five estrildid finch species that differ selectively in grouping behavior (i.e., all five species are monogamous, biparental, and exhibit similar breeding ecologies), and include two species that have independently evolved a highly gregarious and colonial social structure, two species that have independently evolved territoriality, and one species that is modestly gregarious year-round. These studies demonstrate that all three gregarious species exhibit higher densities of OT-like binding sites in the dorsal (pallial) LS than do the territorial species, whereas this pattern tends to reverse in the ventral (subpallial) LS. Notably, the moderately gregarious Angolan blue waxbill (*Uraeginthus angolensis*) exhibits high binding densities in both dorsal and ventral LS subdivisions (Goodson et al., 2009b). A similar binding pattern is observed for a linear V1aR antagonist (Goodson et al., 2006). However, unlike the iodinated OTR antagonist, which appears to bind selectively to the avian OTR, the linear V1aR radioligand appears to be promiscuous (Leung et al., 2011).

These species also differ in VT-ir cell numbers in the BSTm, at least based on standard immunohistochemistry, with the two highly gregarious species having more cells than the moderately gregarious species and the two territorial species (Goodson and Wang, 2006). However, because VT/VP neurons in the BSTm are often weakly immunoreactive and sometimes not visible without the use of colchicine to block axonal peptide transport (Sofroniew, 1985), species differences reported in finches must be interpreted cautiously. In fact, VT-ir cell numbers observed following central colchicine infusions in the violet-eared waxbill [*Uraeginthus granatina*; data collected in conjunction with those reported in Goodson et al. (2012b)] and Angolan blue waxbill (present study) are far greater than reported in (Goodson and Wang, 2006), although only in males. However, relative to zebra finches, these cells are very weakly immunoreactive. In contrast, BSTm VT neurons in zebra finches are more strongly immunoreactive and do not appear to change following infusions of colchicine (Goodson and Kelly, unpubl. obs.).

We here use antisense oligonucleotides to knock down production of VT in the BSTm of male and female Angolan blue waxbills in order to determine whether these species differences in VT anatomy are associated with functional differences between Angolan blue waxbills and zebra finches.

## Materials and methods

### Animals

Experiments were conducted in a humane manner and were in compliance with all federal and institutional regulations. Wild-caught Angolan blue waxbills were obtained from a commercial importer and were housed in mixed-sex cages (10–12 individuals) containing wicker nests. Because it is not possible to sex all blue waxbills based on plumage, the provision of nests allowed us to observe behaviors that aided in the sexing of subjects. Subjects remained in these cages until 5 days prior to pre-testing, at which time they were transferred to same-sex cages. Birds were housed on a 14L:10D photoperiod with full spectrum lighting and were provided finch seed mix, cuttlebone, grit, and water *ad libitum*. Liberal servings of mealworms were also provided. Subjects exhibited modest gonadal recrudescence at the beginning of studies. A total of 31 male and 27 female Angolan blue waxbills were available for the present experiments. Four males and 7 females were used for pilot surgeries and antisense validation, and of these, 1 male and 4 females exhibited accurate cannula placements and were used for the within-subjects validation experiment described below. The remaining birds were used for behavioral experiments, but due to attrition, the final *n*'s for analyses are 21 males and 9 females. Although a portion of this attrition was due to inaccurate cannula placements, we found the blue waxbills (which weigh only ~9 g) to be extremely sensitive to anesthesia, and thus mortality during surgeries was high, particularly for females.

### Surgeries, infusions, and histology

**Figure 1:**
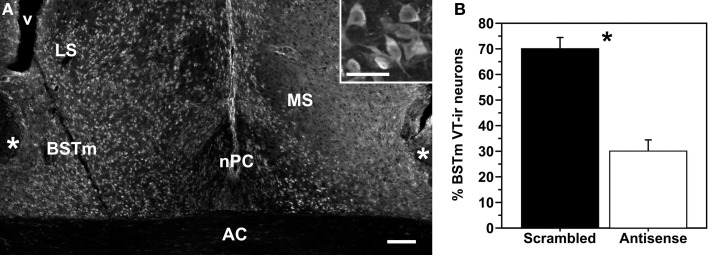
**(A)** Representative knockdown of VT production as shown in a colchicine-treated pilot subject that received infusions of scrambled oligonucleotides in the left hemisphere and VT antisense oligonucleotides in the right hemisphere. *Inset*: Magnified view of VT-ir neurons in the BSTm. Scale bar = 100 μm; 50 μm for inset. Abbreviations: AC, anterior commissure; LS, lateral septum; MS, medial septum; nPC, nucleus of the pallial commissure; v, ventrical. **(B)** Data from a within-subjects validation experiment, showing the percent of total BSTm VT-ir neurons that were observed in hemispheres infused with scrambled (control) and antisense oligonucleotides. ^*^p = 0.01.

Subjects were stereotaxically fitted with a bilateral 26-ga cannula device (1.5 mm tip separation; Plastics One, Akron, OH) aimed at the dorsolateral aspect of the BSTm (see Figure 1A). Cannulae were referenced to the anterior pole of the cerebellum, and were then moved 2.8 mm rostral and advanced 3.0 mm into the brain. Cannulae were affixed to the skull using dental acrylic and veterinary-grade cyanoacrylate glue. The skin was closed with cyanoacrylate glue, and subjects were allowed at least 5 days to recover. Infusions began 2.5 days prior to behavioral testing. Subjects were bilaterally infused at 12 h intervals with either 1 μ g VT antisense oligonucleotides or scrambled oligonucleotides in 0.25 μl of isotonic saline. Injectors extended 1 mm beyond the tip of the guide cannula. Testing was initiated following the 5th infusion (delivered AM) and completed in the morning following the 6th infusion (delivered PM). Subjects were euthanized by an overdose of isoflurane vapor and transcardially perfused with 0.1 M phosphate-buffered saline, followed by 0.4% paraformaldehyde. Brains were post-fixed overnight, transferred to 30% sucrose for 2 days, and then sectioned on a cryostat at 40 μm for verification of cannula placement.

### Antisense validation

The antisense construct used here has previously been validated in zebra finches, using a within-subjects design in which subjects were infused unilaterally into the dorsolateral BSTm with scrambled oligonucleotides, and with antisense oligonucleotides into the contralateral hemisphere (sides counterbalanced across subjects). This design was used because large individual differences in VT-ir cell numbers preclude the use of between-subjects approaches to validation (i.e., by comparing treatment groups). The sequence for the zebra finch VT LNA antisense was CT+CTGC CAT GG+CT+CA, and the sequence for the zebra finch VT LNA scrambled oligonucleotide construct was AG+C GTA TCT TG+CC+CC (see GenBank accession number ABQF01053428 for sequence data on the gene encoding zebra finch VT). In this previous experiment, antisense infusions produced an average reduction of VT-ir neuron numbers by 55% relative to the control hemisphere (Kelly et al., 2011).

We here used this same within-subjects design to verify efficacy of the VT antisense oligonucleotides in the Angolan blue waxbill, using 4 males and 7 females in which we piloted BSTm coordinates, of which 1 male and 4 females exhibited accurate placements in the dorsolateral BSTm. Following the 5th infusion of oligonucleotides (when behavioral testing would normally occur), subjects were infused with colchicine (3% in 0.25 μl saline) to block axonal transport of peptide, increasing our ability to visualize VT-producing cells. Histology was conducted as described above, and tissue was immunofluorescently labeled for VT as previously described (Kelly et al., 2011) using a rabbit VP antibody (ImmunoStar, Hudson, WI).

### Behavioral testing

Group size preference (gregariousness) and social contact were quantified in a two-choice paradigm. In this test, subjects were placed into a 1 m wide cage that was divided into 7 zones by perches. The perches at each end of the cage were approximately 4 cm from the cage wall, and were adjoined by a 0.5 m wide cage containing 2 stimulus birds at one end and 10 stimulus birds at the other (sides counterbalanced across subjects). Subject location was recorded every 15 s for 4 min [see Goodson et al. (2009b); Kelly et al. (2011)], with sides changed at 2 min. “Social contact” was operationally defined as the percent of test time that the subject spent in the two zones closest to the stimulus cages combined, and “gregariousness” was operationally defined as the percentage of that contact time that was spent next to the larger group.

Anxiety-like behavior was assessed using tests of novelty-suppressed feeding and exploration. For novelty-suppressed feeding, food was removed from the subjects' cage prior to lights-on, and 10 min after lights-on subjects were placed into a small, unfamiliar test cage (31 cm W × 20 cm H × 36 cm D) with a novel object (a purple nitrile glove) placed above a food dish. The latency to feed was quantified during a 90 min video-recorded trial. For exploration tests, subjects were placed into an unfamiliar cage (1.3 m W × 0.43 m H × 0.36 m D) containing 3 clusters of tree branches, and the number of hops/flights and number of visits to the branch clusters were recorded during a 3 min period.

### Statistics

With the exception of antisense validation data, which were analyzed by paired *t*-tests, data were not normally distributed and were therefore analyzed using Mann-Whitney tests, comparing subjects that received scrambled oligonucleotides and subjects that received antisense oligonucleotides, with data presented as post-testing minus pre-testing values. Because antisense knockdown of BSTm VT production produces sex-specific effects on social behavior in zebra finches (Kelly et al., 2011; Kelly and Goodson, 2013), data for gregariousness and social contact were analyzed separately for each sex. However, because VT knockdown increases anxiety in both male and female zebra finches (Kelly et al., 2011), anxiety data were analyzed for the sexes combined as well as separately.

## Results

### Antisense validation

As shown in Figure 1, pilot subjects infused unilaterally with scrambled oligonucleotides and contralaterally with antisense oligonucleotides exhibit significantly fewer VT-ir neurons in the BSTm of the antisense-treated hemisphere (*t* = 4.61; *p* = 0.01; *n* = 5), with a mean reduction of ~57%. The photo in Figure 1 is from a colchicine-infused male subject, and as can be seen, large numbers of weakly immunoreactive VT neurons are observed in the BSTm and other areas as well. These cells extend into the LS, lateral BST, medial telencephalon (along the lateral ventricle), and hippocampus, extending as far rostral as the nucleus accumbens. This is not observed in females, which show only scattered VT-ir neurons in the BSTm following colchicine infusions. Importantly, we have observed this sex difference in a much larger sample of tissue from another *Uraeginthus* species, the violet-eared waxbill, collected in conjunction with an experiment focused on vasoactive intestinal polypeptide, in which all subjects were treated with colchicine (Goodson et al., 2012b). Virtually all of the 11 males in that study showed extensive distributions of VT-ir neurons as just described, whereas the 10 females showed either no VT-ir neurons in extrahypothalamic areas outside of the BSTm, or very few in relation to males (Goodson and Kelly, unpubl. obs.).

### BSTm VT antisense knockdown reduces social contact, but has no effect on gregariousness

**Figure 2:**
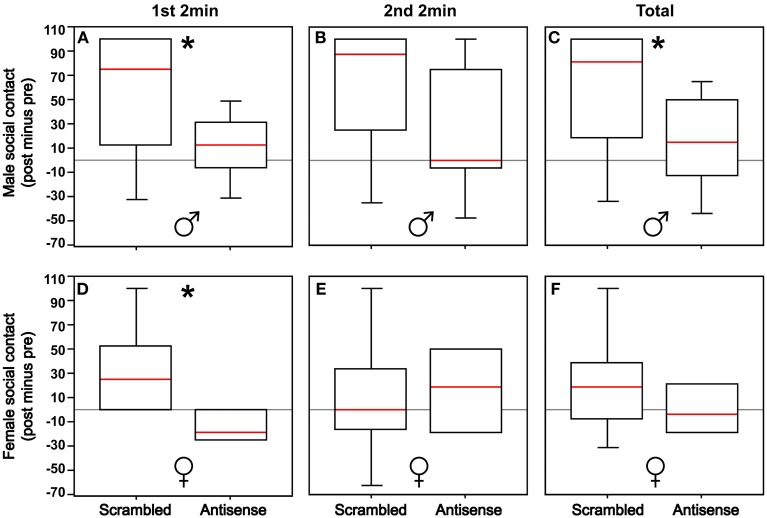
**(A,B)** Knockdown of VT production in the BSTm reduces social contact in males in the first 2 min of testing (^*^p = 0.03), but not in the second 2 min of testing following the rotation of the stimulus cages containing 2 or 10 same-sex birds (*p* = 0.13). **(C)** With the first and second test periods combined, there is an overall reduction in social contact in males (^*^p = 0.04). **(D,E)** Knockdown of VT production in the BSTm reduces social contact in females in the first 2 min of testing (*p* = 0.04), but not in the second 2 min of testing (*p* = 0.54). **(F)** With the first and second test periods combined, there is no overall effect on social contact in females (^*^p = 0.46). Data are shown as post-treatment minus pre-surgical values. Box plots show the median (red line), 75th and 25th percentile (box), and 95% confidence interval (whiskers).

Males infused with VT antisense oligonucleotides exhibited less social contact compared to control males (Mann-Whitney *U* = 25.5; *p* = 0.04). Effects were more pronounced in the first 2 min of testing (Mann-Whitney *U* = 24.5; *p* = 0.03) relative to the second 2 min after the stimulus cages containing large and small conspecifics were rotated (Mann-Whitney *U* = 33.0; *p* = 0.13; Figures 2A–C). In contrast, antisense infusions produced no overall effect on social contact time in females (Mann-Whitney *U* = 7.0; *p* = 0.46), although antisense did reduce social contact in the first 2 min of testing (Mann-Whitney *U* = 2.0; *p* = 0.04), but not in the second 2 min (Mann-Whitney *U* = 7.5; *p* = 0.54; Figures 2D–F).

We observed no significant effects of VT antisense infusions on gregariousness in males or females regardless of whether the data were analyzed with the first 2 min and second 2 min pooled or separately (all *p* > 0.17). However, these analyses were of limited power due to empty cells, resulting from the fact that numerous subjects did not exhibit contact time with the large group in pre-testing, post-testing, or both (*n* = 4 females and *n* = 12 males eliminated from the analysis). Thus, we reanalyzed data for gregariousness by adding the second zone at each end of the test cage, effectively increasing the contact zone, and thus increasing the number of subjects included in the analysis (*n* = 8 scramble control males, *n* = 8 antisense males, *n* = 4 scramble control females, *n* = 4 antisense females). This modified analysis yielded no significant effects (all *p* > 0.08).

### BSTm VT neurons promote social contact in a sexually differentiated manner

**Figure 3:**
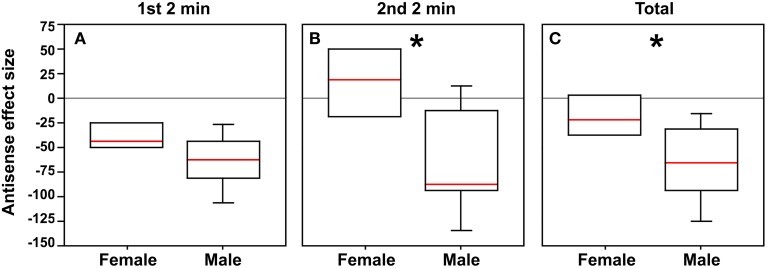
**In order to determine whether male and female subjects differ in their response to antisense infusions, the median social contact score (post-minus pre-surgical values, as shown in Figure 2) of scrambled control subjects was subtracted from the score of each subject that received antisense, yielding a measure of “antisense effect size.” (A)** Although there is no significant difference between sexes during the first 2 min, strong differences are observed for the second 2 min (^*^p = 0.03; **B)** and for the total test period (^*^p = 0.05; **C)**.

Although antisense infusions produced significant effects on social contact in both males and females as described above, the effects in males appeared to be much more pronounced. Thus, in order to determine whether male and female subjects differed in their response to antisense infusions, we subtracted the median social contact score of scrambled control subjects from the score of each subject that received antisense (performed separately for males and females). The resultant “antisense effect size” did not differ between males and females in the first 2 min (Mann-Whitney *U* = 11; *p* = 0.11), but did differ in the second 2 min after rotation of the stimulus cages (Mann-Whitney *U* = 6.5; *p* = 0.03), and for the total 4 min test period (Mann-Whitney *U* = 8; *p* = 0.05; Figures 3A–C).

### BSTm VT antisense knockdown has no effect on anxiety-like behavior

Subjects that received VT antisense oligonucleotides did not differ from subjects that received scrambled oligonucleotides in their latency to feed in the novelty-suppressed feeding paradigm or for exploration measures in the novel environment (all *p* > 0.15 regardless of whether data were analyzed with sexes separated or combined).

## Discussion

### Species differences in VT neuronal function

In most tetrapod vertebrates, VT/VP immunoreactivity in the BSTm and LS is expressed only during the breeding season (Goodson and Bass, 2001; De Vries and Panzica, 2006), suggesting that the primary functions of this circuitry are tied to reproduction. Indeed, BSTm VT/VP neurons exhibit strong responses to sexual stimuli in male mice, chickens, and finches (Goodson et al., 2009a; Ho et al., 2010; Xie et al., 2011), and knockdown of BSTm VT production profoundly reduces courtship singing in male zebra finches while concomitantly increasing aggression (Kelly and Goodson, 2013). Similarly, VT-ir cells are more numerous in male zebra finches that reliably court females than males that fail to court females (Goodson et al., 2009a). However, in contrast to seasonally breeding species, several estrildid finch species that breed semi-opportunistically do not exhibit temporal fluctuations in VT immunoreactivity (Kabelik et al., 2010), and thus VT circuitry arising in the BSTm of these species is available to modulate non-reproductive affiliation behaviors as well. In fact, antisense experiments show that this circuitry modulates same-sex gregariousness (i.e., non-reproductive affiliation) in male but not female zebra finches (Kelly et al., 2011; Kelly and Goodson, 2013). In contrast, we here show that BSTm VT cells do not modulate gregariousness in the less gregarious Angolan blue waxbill, but do promote social contact, most strongly in males. Furthermore, whereas knockdown of BSTm VT production produces robust increases in anxiety in both male and female zebra finches (Kelly et al., 2011; Kelly and Goodson, 2013), no effects on anxiety are observed for the Angolan blue waxbill.

These species differences may be interpreted in multiple ways. First, because zebra finches typically form very large groups (Zann, 1996), we hypothesize that redundant neural systems have evolved to ensure that individuals in this species always maintain social contact. If this is so, then knockdown of VT production may not be sufficient to reduce social contact in zebra finches (i.e., due to redundant neural systems), but is sufficient to reduce preferences for larger groups, while in the less social blue waxbill it reduces social contact altogether. By this interpretation, BSTm VT neurons may promote affiliation similarly in both zebra finches and blue waxbills, but with species-specific effects due to interactions with other neural systems that modulate social behavior. A problem for this interpretation is that whereas knockdown of BSTm VT production reduces gregariousness in male zebra finches by ~80%, social contact is actually *increased*, albeit only modestly (~25%) (Kelly et al., 2011). This observation suggests that gregariousness and social contact are not on a single continuum, although interpretation of the social contact data in zebra finches is difficult (and the relevant finding may be anomalous), given that central infusions of OTR and V1aR antagonists have no detectable impact on social contact, yet produce profound reductions in gregariousness (Goodson et al., 2009b; Kelly et al., 2011). Hence the hypothesis that BSTm VT neurons promote affiliation similarly in zebra finches and waxbills cannot be rejected with confidence.

Whereas the above interpretation places the divergent antisense effects in finches and waxbills on a continuum, an alternative possibility is that the divergent effects are truly qualitative and rooted in significant species differences in VT anatomy. As discussed earlier, gregarious estrildid species (including zebra finches and Angolan blue waxbills) exhibit higher densities of OT-like binding sites in the dorsal (pallial) LS than territorial species, where this pattern tends to reverse in the ventral (subpallial) LS. However, unlike zebra finches, Angolan blue waxbills exhibit high binding densities in both dorsal and ventral LS subdivisions (Goodson et al., 2009b). Similar binding differences are observed for linear V1aR antagonist (Goodson et al., 2006), although this antagonist does not appear to bind selectively to the avian V1aR (Leung et al., 2011). As previously shown using standard immunocytochemistry, zebra finches also exhibit many more VT-ir neurons in the BSTm than do Angolan blue waxbills, and these cells tend to be much more strongly immunoreactive (Goodson and Wang, 2006). However, as shown here, blue waxbills treated with colchicine exhibit larger numbers of weakly immunoreactive neurons in the BSTm than previously reported, and at least in males this cell group is very large and extends into adjacent areas such as the LS and lateral BST. In contrast, colchicine infusions do not alter the basic pattern of immunolabeling in zebra finches (Goodson and Kelly, unpubl. obs.). Given these significant species differences in VT anatomy and receptor distributions, the differential social effects of VT knockdown in zebra finches (reduction of gregariousness) and Angolan blue waxbills (reduction of social contact) may reflect truly qualitative variation in the way in which VT modulates behavior in these species.

The hypothesis that differences in VT anatomy are reflected in qualitative differences in behavioral function is strongly supported by the findings that whereas antisense knockdown of BSTm VT production dramatically increases anxiety-like responses to novelty in both male and female zebra finches (Kelly et al., 2011; Kelly and Goodson, 2013), antisense infusions have no discernable impact on anxiety-like behavior in the blue waxbill. Studies in rodents likewise suggest an involvement of septal VP in anxiety that is species- and/or context-specific, although it remains to be determined whether these effects are attributable to VP release from neurons in the BSTm. For example, in individually housed male Wistar rats, septal infusions of V1aR antagonists and V1aR antisense reduce anxiety in the elevated plus maze (EPM), suggesting that endogenous VT release into the septum is anxiogenic (Landgraf et al., 1995; Liebsch et al., 1996). Interestingly, another study in *group-housed* male Wistar rats showed that retrodialysis of VP into the septum increases activity in the open arms of the EPM, suggesting that septal VP is *anxiolytic* (Appenrodt et al., 1998). Hence, it is possible that social housing conditions (individual vs. group) may modulate the relationship between septal VP release and anxiety. Findings in lab mice are likewise mixed. For instance, re-expression of V1aR in the LS of V1aR knockout mice does not alter anxiety-like behavior in the EPM, light/dark box, or open field tests, although overexpression of V1aR in the LS of wild type mice does weakly increase anxiety-like behavior, but only in the light/dark box (Bielsky et al., 2005). Hence, endogenous VP release into the LS likely exerts little or no effects on anxiety in male mice, but modulates anxiety in male rats in a manner that appears to vary in relation to social and/or other contextual factors (which may be lab-specific). Combined with the findings from zebra finches and Angolan blue waxbills, it is clear that VT/VP modulates anxiety-like behavior in very species-specific (and likely context-specific) ways, although it remains to be determined whether these differential effects on anxiety are integrated with the expression of social behavior.

### Sex differences in VT neuronal function

In most tetrapod vertebrate species, males exhibit significantly more VT/VP neurons in the BSTm than do females and significantly denser projections to basal forebrain areas such as the LS (Goodson and Bass, 2001; De Vries and Panzica, 2006), although this sexual dimorphism is quite subtle in zebra finches (Kabelik et al., 2010), even following colchicine infusions (Goodson and Kelly, unpubl. obs.). Similarly, no sex differences in VT-ir cell numbers have been detected in several other estrildid finch species, including two in the genus *Uraeginthus*—the Angolan blue waxbill and violet-eared waxbill (Goodson and Wang, 2006). However, violet-eared waxbills treated with colchicine as part of another experiment (Goodson et al., 2012b) show profound sex differences in VT-ir cell numbers in the BSTm and other extrahypothalamic areas (Goodson and Kelly, unpubl. obs.), and limited data from the present experiments demonstrate a comparable condition in the Angolan blue waxbill

Although zebra finches show only modest dimorphism in VT anatomy (Kabelik et al., 2010), antisense experiments reveal large sex differences in function. For instance, knockdown of VT production in the BSTm reduces preferences for the larger of two groups (gregariousness) by 80% in males while having no effect in females, and produces large male-specific increases in aggression (Kelly et al., 2011; Kelly and Goodson, 2013). In contrast, the differential effects of VT knockdown in male and female waxbills are quantitative in nature, not dichotomous. The combined findings therefore suggest that the degree of sex differences in function is not wholly dependent upon the degree of sexual dimorphism in VT anatomy. Sex differences in receptor distributions could potentially account for this surprising result, although no sex differences have been described to date in finches (Goodson et al., 2006, 2009b). Fos responses to simple social stimuli are also similar in male and female zebra finches (Goodson and Wang, 2006), but a recent study using more complex stimuli shows that BSTm VT neurons exhibit Fos responses in zebra finches that vary not only across sexes, but across different personality types as well (Kelly and Goodson, unpubl. obs.). Hence, sex differences in VT function may rely on a combination of sex-specific neuronal responses and sexually differentiated VT production. Given that BSTm VT neurons are strongly regulated by steroid hormones (Goodson and Bass, 2001; De Vries and Panzica, 2006), sex differences in VT response and function may be hormone-dependent.

Finally, the fact that male waxbills express extrahypothalamic VT-ir cells not only the BSTm, but also in adjacent areas, suggests that antisense effects in males may rely to some extent on VT knockdown in areas such as the LS and lateral BST [although importantly, BSTm infusions do not impact VT production in the paraventricular hypothalamus; (Kelly et al., 2011)]. However, the lack of VT production in these additional sites in females clarifies the situation considerably, given that antisense effects in female Angolan blue waxbills are qualitatively similar to males, albeit weaker. Thus, it is clear that VT neurons in the BSTm promote social contact in Angolan blue waxbills, although the possible contributions of cells outside of the BSTm in males cannot be excluded.

## Conclusions

Anatomical features of VT/VP circuits arising in the BSTm vary across species, suggesting that BSTm VT neurons may exert species-specific effects on behavior. We here show that knockdown of VT production in the BSTm reduces social contact in the modestly gregarious Angolan blue waxbill, without effects on anxiety-like behavior or group size preferences (gregariousness). Effects are observed in both sexes, but are stronger in males. In contrast, VT knockdown in the highly social zebra finch dramatically increases anxiety-like behavior in both males and females and produces a profound male-specific decrease in gregariousness that is accompanied by a much more modest increase in social contact (Kelly et al., 2011; Kelly and Goodson, 2013). These species differences may be rooted in one or more factors, including species differences in VT production, VT receptor distributions, VT interactions with other neurochemical systems, and/or patterns of VT neuronal response to environmental stimuli. However, despite the presence of species differences, BSTm VT neurons appear to generally increase aspects of affiliation (including courtship), particularly in males.

### Conflict of interest statement

The authors declare that the research was conducted in the absence of any commercial or financial relationships that could be construed as a potential conflict of interest.
